# CXCR2 modulates bone marrow vascular repair and haematopoietic recovery post‐transplant

**DOI:** 10.1111/bjh.13335

**Published:** 2015-03-09

**Authors:** Sarah J. M. Hale, Ashley B. H. Hale, Youyi Zhang, Dominic Sweeney, Nita Fisher, Mark van der Garde, Rita Grabowska, Emma Pepperell, Keith Channon, Enca Martin‐Rendon, Suzanne M. Watt

**Affiliations:** ^1^ Stem Cell Research LaboratoryNHS Blood and TransplantJohn Radcliffe HospitalOxfordUK; ^2^ Nuffield Division of Clinical and Laboratory SciencesRadcliffe Department of MedicineUniversity of OxfordJohn Radcliffe HospitalOxfordUK; ^3^ Cardiovascular MedicineRadcliffe Department of MedicineUniversity of OxfordJohn Radcliffe HospitalOxfordUK

**Keywords:** bone marrow transplantation, stem cell niche, bone marrow vascular niche, CXCL8, CXCR2

## Abstract

Murine models of bone marrow transplantation show that pre‐conditioning regimens affect the integrity of the bone marrow endothelium and that the repair of this vascular niche is an essential pre‐requisite for successful haematopoietic stem and progenitor cell engraftment. Little is known about the angiogenic pathways that play a role in the repair of the human bone marrow vascular niche. We therefore established an *in vitro* humanized model, composed of bone marrow stromal and endothelial cells and have identified several pro‐angiogenic factors, VEGFA, ANGPT1, CXCL8 and CXCL16, produced by the stromal component of this niche. We demonstrate for the first time that addition of CXCL8 or inhibition of its receptor, CXCR2, modulates blood vessel formation in our bone marrow endothelial niche model. Compared to wild type, *Cxcr2* ^−/−^ mice displayed a reduction in bone marrow cellularity and delayed platelet and leucocyte recovery following myeloablation and bone marrow transplantation. The delay in bone marrow recovery correlated with impaired bone marrow vascular repair. Taken together, our data demonstrate that CXCR2 regulates bone marrow blood vessel repair/regeneration and haematopoietic recovery, and clinically may be a therapeutic target for improving bone marrow transplantation.

Haematopoietic stem and progenitor cells (HSPCs) are responsible for the generation and maintenance of all the blood cells in the body. In adults, the major site of haematopoiesis is the bone marrow, a structure which houses cellular components that form specialized micro‐environmental niches, which tightly control the quiescence, self‐renewal and differentiation of HSPCs (Wolf & Trentin, 1968; Schofield, 1978; Wilson & Trumpp, 2006; Sacchetti *et al*, 2007; Morrison & Spradling, 2008; Sugiyama & Nagasawa, 2012; Ding & Morrison, 2013). Studies in mice have demonstrated that pre‐conditioning regimes prior to bone marrow transplantation (BMT) can significantly affect the integrity of the bone marrow vasculature and that vascular repair of this niche following pre‐conditioning is an essential pre‐requisite for successful HSPC engraftment (Rafii *et al*, 2003; Avecilla *et al*, 2004; Kopp *et al*, 2005; Hooper *et al*, 2009; Butler *et al*, 2010; Kobayashi *et al*, 2010). Slayton *et al* (2007) also showed that the regenerated bone marrow sinusoidal endothelium following pre‐conditioning is host‐derived. These studies highlight the importance of the vascular niche in BMT and warrant further research focused on modulating the repair of this niche for therapeutic intervention.

Although a role for positive regulators of murine bone marrow angiogenesis, such as Angiopoietin 1 (ANGPT1) and vascular endothelial growth factor A (VEGFA), has started to emerge (Avecilla *et al*, 2004; Kopp *et al*, 2005; Hooper *et al*, 2009), studies on the human bone marrow endothelial niche have been hampered by a lack of suitable tissue‐specific *in vitro* models. Chemokine (C‐X‐C motif) ligand 8 (CXCL8), a chemokine produced by various cells, is widely associated with inflammation and neutrophil recruitment, but is also a potent human pro‐angiogenic factor (Heidemann *et al*, 2003; Kimura *et al*, 2011; Singh *et al*, 2011; Roubelakis *et al*, 2013). Additionally, the CXCL8/chemokine (C‐X‐C motif) receptor 2 (CXCR2) pathway can be targeted to inhibit tumour angiogenesis (Singh *et al*, 2010; Nannuru *et al*, 2011). However, the role of this chemokine and receptor in supporting bone marrow angiogenesis and haematopoietic recovery following myeloablation is novel and has not been previously investigated.

In this study, we found that the CXCL8/CXCR2 pathway modulated bone marrow angiogenesis and therefore haematopoiesis. These results may be relevant both clinically and in strategies aimed at improving HSPC reconstitution.

## Methods

### Cells

Bone marrow endothelial cells (BMEC‐60, subsequently referred to as BMEC) were obtained from Professor E van der Schoot (Rood *et al*, 2000). These cells and human umbilical vein endothelial cells (HUVEC; Lonza, Cambridge, UK) were cultured in Endothelial Cell Growth Medium‐2 (EGM‐2; Lonza) according to the manufacturer's instructions. In some experiments endothelial cells were labelled with a lentiviral vector system encoding eGFP (a kind gift from Professor Adrian Thrasher, Institute of Child Health, London) at a multiplicity of infection of 4, as previously described (Roubelakis *et al*, 2013). Human bone marrow stromal cells (hBMSC; catalogue number 2M‐302) and primary human dermal fibroblasts (hDF, catalogue number CC‐2511; CD90^+^, CD105^+^, CD45^−^, CD31^−^, CD34^−^, CD14^−^) were obtained from Lonza and cultured in Myelocult medium (Stem Cell Technologies, Cambridge, UK) supplemented with hydrocortisone (10^−6^ mol/l) or Dulbecco's modified Eagle's medium supplemented with 10% (v/v) fetal calf serum (FCS) respectively, and used at passage 3–5. Human umbilical cord blood (UCB) units were collected from the John Radcliffe Hospital, Oxford, with informed consent and ethical permission. HSPCs were purified using the Magnetic‐activated cell sorting (MACS) CD133 cell isolation kit (Miltenyi Biotec, Bisley, UK) as described previously (Pepperell & Watt, 2013).

### Bone marrow endothelial niche assay

Human bone marrow stromal cells or hDF and BMEC or HUVEC were co‐cultured at various cell ratios in bio‐coat plates (BD Biosciences, Oxford, UK) for up to 14 d in complete EGM‐2 supplemented with 8% (v/v) FCS. hDF and HUVEC were co‐cultured as previously described (Khoo *et al*, 2011; Zhou *et al*, 2012). Complete media changes were performed every 2–3 d. In some experiments CXCL8 or neutralizing antibodies were added to the co‐cultures every 2–3 d at concentrations indicated in the figure legends. Co‐cultures were fixed with 4% (w/v) paraformaldehyde (PFA) and stained with CD31 antibody as described previously (Zhang *et al*, 2009), or monitored directly for eGFP expression. Images of vessel networks were captured using a fluorescence microscope (TE2000‐U; Nikon Ltd., Kingston upon Thames, UK) at ×4 magnification. Images were recorded using the Simple PCI, version 6.6.0.0 software (Hamamatsu Corporation, Welwyn Garden City, UK) and then processed using Adobe Photoshop CS2 9.0.2 (Reindeer Graphics, Asheville, NC, USA). Vessel networks were analysed using the Angiosys 1.0 software (TCS Cell Works, Buckingham, UK) as described elsewhere (Zhou *et al*, 2012). For co‐culture experiments with HSPCs, BMEC or HUVEC and hBMSC was first co‐cultured in complete EGM‐2 containing 8% (v/v) FCS for a period of 12–14 d before treatment with 10 μg/ml mitomycin C (Sigma‐Aldrich Ltd., Gillingham, UK) for 1 h. Cells were then washed prior to addition of 5000 HSPCs/well. Co‐cultures were then maintained in Myelocult medium (Stem Cell Technologies) supplemented with 10^−6^ mol/l hydrocortisone without cytokine supplementation.

### Transwell migration assay

Bone marrow endothelial cells were plated on to 8‐μmol/l 24‐well plate transwell filters (BD Biosciences) and left to adhere overnight in complete EGM‐2. Next day, the lower wells were replaced with complete EGM‐2 supplemented with either 100 ng/ml CXCL8 (Peprotech, London, UK) and incubated for a further 24 h. Post‐migration cells remaining on the upper part of the transwell were carefully removed with a cotton bud. Filters were then removed from the housing and mounted onto slides with fluorescent mounting media containing 4′,6‐diamidino‐2‐phenylindole (DAPI; Vector Laboratories, Peterborough, UK). Migrated cells were viewed at ×60 magnification using a fluorescent microscope (TE2000‐U; Nikon Ltd.) and 10 randomly selected fields per transwell were counted manually.

### Flow cytometry

For flow cytometric analysis of HSPCs, suspension cells were harvested, treated with FcR blocking reagent (Miltenyi Biotec) and stained with CD45‐peridinin chlorophyll‐cyanin 5.5 (CD45‐PerCP‐CY5.5; mIgG1, Clone TU116; BD Biosciences), or CD34‐allophycocyanin (CD34‐APC; mIgG2a, Clone AC136) and CD133‐phycoerythrin (CD133‐PE; mIgG2b, Clone AC133) antibodies or matched isotype control antibodies (Miltenyi Biotec). For analysis of hBMSC, cells were trypsinized and then stained with either CD31‐PE (mIgG1, Clone WM9), CD44‐PE (mIgG1, Clone 515), CD14‐PE‐CY7 (mIgG2a, Clone M5E2), CD29‐PE (mIgG1, Clone MAR4), CD45‐PerCP (mIgG1, Clone 2D1; all BD Biosciences), CD90‐fluorescein isothiocyanate (CD90‐FITC; mIgG1, 5E10), CD105‐FITC (mIgG1, 166707), CD166‐PE (mIgG1, Clone 105902; all R&D Systems, Abingdon, UK) or CD34‐APC (mIgG2a, AC136; Miltenyi Biotech) antibodies or matched isotype controls from the same manufacturer. For analysis of bone marrow endothelium, mouse bones were harvested 4 d post‐irradiation, crushed and then stained with a panel of haematopoietic/endothelial markers exactly as described previously by Hooper *et al* (2009). In these experiments DAPI was used to distinguish dead and viable cells. Data were acquired using a BD LSR II flow cytometer and analysed using the FACS Diva software (BD Biosciences).

### Angiogenic protein analysis

Conditioned media (CM) were collected from hBMSC or hDF after culturing them in EGM‐2 for 48 h. Human Angiogenesis Antibody Arrays (R&D systems) were performed as per the manufacturer's instructions and analysed using ImageJ analysis software (http://imagej.nih.gov/ij/) to determine mean pixel density (MPD) (Roubelakis *et al*, 2013). The relative MPD for each protein tested was calculated as a percentage of the positive assay control after subtraction of the negative control MPD. Specific cytokines were quantified using Quantikine enzyme‐linked immunosorbent assay (ELISA) kits according to the manufacturer's instructions (R&D Systems).

### Endothelial tubule formation assay

eGFP‐labelled BMEC were plated onto growth factor reduced Matrigel as described previously (Khoo *et al*, 2011). Cytokines or carrier control was added to the wells at the concentrations indicated in the figures. For blocking studies, anti‐human CXCR2 neutralizing antibody (R&D systems, clone 48311) was added together with recombinant human CXCL8 (Peprotech). Cells were incubated at 37°C for 16 h and eGFP^+^ vessel networks were captured directly at ×10 magnification using a fluorescence microscope (TE2000‐U; Nikon Ltd.). Images were recorded using the Simple PCI version 6.6.0.0 software and then processed using Adobe Photoshop CS2 9.0.2. Vessel networks were then analysed using the Angiosys 1.0 software.

### Immunohistochemistry of cultured cells and bone marrow

Cells were fixed with PFA and permeabilized (Triton ×100) before incubation with anti‐human CXCL12 (Abcam, Cambridge, UK; rabbit polyclonal), anti‐human SCF (Abcam; rabbit polyclonal), anti‐human CXCR2 (R&D systems, clone 48311), anti‐human CXCL8 (eBiosciences, Hatfield, UK; clone NAPII) or isotype controls from the same manufacturer. Anti‐mouse or rabbit Alexafluor 555 secondary antibody were used for detection (Invitrogen Ltd., Paisley, UK). Images were taken at ×40/60 magnification using a fluorescence microscope (TE2000‐U; Nikon Ltd.), captured using Axiovision software (Carl Zeiss Microscopy Ltd, Cambridge UK) and then processed using Adobe Photoshop CS2 9.0.2. Mouse bone marrow endothelial staining was performed as previously described (Hooper *et al*, 2009). Type I and II sinusoidal vessels were quantified at ×60 magnification by two independent researchers using at least five random fields per femur. Images were taken using an inverted light microscope (TE600; Nikon Ltd.) and captured using Axiovision software. Paraffin‐embedded human reactive bone marrow trephine biopsies were obtained from the Department of Cellular Pathology at the John Radcliffe Hospital, Oxford, UK, then sectioned, deparaffinized, rehydrated and antigen retrieval performed. Samples were blocked with 5% (v/v) bovine serum albumin and 10% (v/v) normal goat serum (both Sigma‐Aldrich), before incubation with anti‐human CD31 antibody (clone WM59; AbD Serotec, Kidlinton, UK) and anti‐human CXCR2 antibody (clone 48311; R&D Systems) or isotype controls overnight at 4°C. Staining was visualized using anti‐mouse IgG1 conjugated to Alexa 555 and anti‐mouse IgG2a conjugated to Alexa 488 for 1 h at room temperature (Life Technologies, Paisley, UK). Sections were mounted in SlowFade Gold anti‐fade reagent with DAPI (Life Technologies) before visualization by fluorescence microscopy (TE600; Nikon Ltd.). Images were captured using Axiovision software.

### Animals and transplantation studies

Mice were housed in individually ventilated cages and handled using aseptic techniques. Male and female B6.129S2(C)‐*Cxcr2* ^*tm1Mwm*^/J mice, heterozygous for *Cxcr2* were obtained from Jackson Laboratories (Bar Harbor, ME, USA) and bred in‐house. Twelve 16‐week‐old, age‐ and sex‐matched littermates were treated prophylactically with antibiotics (Septrin; 1 mg/ml drinking water) for 7 d prior to and during the entire duration of the transplantation studies. Animals were treated with a total of 9·5 Gy, administered by split dose, prior to a single intravenous injection of 1 × 10^6^ wild type mouse bone marrow. Blood samples were collected into heparinized capillary tubes (Fisher Scientific, Loughborough, UK) at days 4, 7, 10 and 14 post‐irradiation. Bone marrow was collected at days 4 and 14 post‐irradiation (as indicated in the figure legends) and either fixed and sectioned as above, or by crushing sets of tibias and fibias using a pestle and mortar and then graded needles and syringes to generate a single cell suspension. Blood and bone marrow analysis was performed using a veterinary ABC analyser using a mouse species analysis card (Horiba, Northampton, UK). All animal experiments were reviewed and approved by the University of Oxford Animal Ethical Review Committee and conducted under the authority of the relevant UK Home Office approved licences.

### Statistics

Data are presented as means ± standard deviation from at least three independent experiments with statistical significance calculated using the Student's *t*‐test. *P* values ≤ 0·05 were considered statistically significant.

## Results

### Human bone marrow endothelial cells form quantifiable vessel networks when co‐cultured with human bone marrow stromal cells and maintain haematopoietic progenitor cells *in vitro*

**Figure 1:**
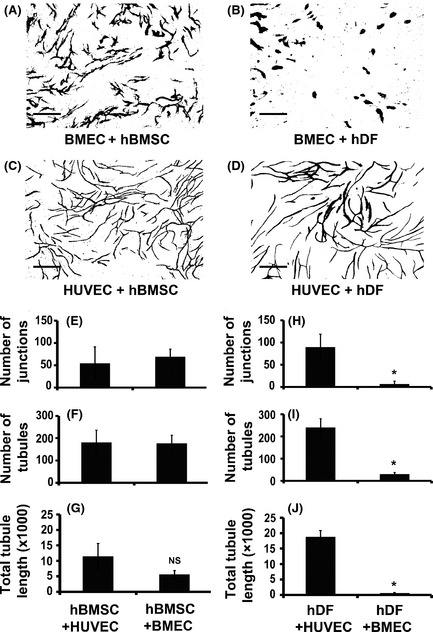
Human bone marrow stromal cells but not human dermal fibroblasts (hDF) support tubule formation of bone marrow endothelial cells. Human bone marrow stromal cells (hBMSC) or hDF were co‐cultured with either human bone marrow endothelial cell (BMEC) or human umbilical vein endothelial cells (HUVEC) for 14 d in complete Endothelial Cell Growth Medium‐2 supplemented with 8% (v/v) fetal calf serum. Vessel networks were visualized by staining with CD31 antibody. Representative images of vessel formation (scale bars 500 μm) are shown from co‐cultures of BMEC with hBMSC (A), BMEC with hDF (B), HUVEC with hBMSC (C) and HUVEC with hDF (D) at optimal cell ratios (6 × 10^3^ BMEC/HUVEC: 4 × 10^4^ hBMSC and 1·5 × 10^3^ BMEC/HUVEC: 1 × 10^4^ hDF). Quantification of number of junctions (branch points; E), number of tubules (F) and total tubule length (G) for HUVEC or BMEC co‐cultured with hBMSC and number of junctions (H), number of tubules (I) and total tubule length (J) for HUVEC or BMEC co‐cultured with hDF are shown. Results were analysed using Student's *t*‐test, *P ≤ 0·05. Data are mean ± standard deviation of at least three separate experiments using at least two separate batches of hDF and hBMSC.

We set out to establish a humanized bone marrow endothelial niche model by co‐culturing primary hBMSC with human bone marrow endothelial cells (BMEC). At various BMEC:hBMSC cell ratios all generated recognizable vascular structures *in vitro* (Figure S1A). In order to determine whether the ability of BMEC to form tubule networks *in vitro* depended on bone marrow *versus* non‐bone marrow stromal cells, we co‐cultured BMEC and hBMSC or hDF, using HUVEC as a positive control. Figure 1 shows representative images and quantification of the optimal vessel formation achieved when co‐culturing BMEC or HUVEC with either hBMSC or hDF at the optimal endothelial:stromal cell ratio. hBMSC supported vessel formation of both BMEC and HUVEC (Fig 1A and C). Surprisingly, after testing a wide range of endothelial:hDF co‐culture ratios and several batches of primary hDF, we consistently found that they failed to support significant vessel network formation of BMEC (Fig 1B), whilst hDF supported robust vessel formation of HUVEC (Fig 1D). These data indicate that hBMSC supported vessel formation of both BMEC and HUVEC, with no statistically significant difference (*P* = 0·08) in the number of junctions (branch points) (Fig 1E) or tubules (Fig 1F) obtained. Although we noted that co‐culture of BMEC with hBMSC seemed to result in shorter total tubule length, than those of HUVEC co‐cultures with hBMSC, this did not quite reach statistical significance (Fig 1G; *P* = 0·06). In contrast, in co‐culture with BMEC, hDF supported significantly less vessel formation, with 12‐fold fewer junctions (Fig 1H), 7·5‐fold shorter tubules (Fig 1I) and 26‐fold less total tubules (Fig 1J), than HUVEC co‐cultured with hDF (*P* ≤ 0·05). Taken together, these data indicate that hDF are unable to support significant vessel formation of BMEC.

Phenotypic analysis of hBMSC by flow cytometry revealed that whilst these cells lack expression of haematopoietic and endothelial markers (e.g. CD31, CD45, CD34), they do express typical mesenchymal/fibroblastic markers, such as CD166, CD44, CD29, CD105 and CD90, at high levels (>87%; Figure S1B), thus confirming that hBMSC displayed a similar phenotype to that of hDF and mesenchymal stem cells (Blasi *et al*, 2011).

In order to confirm that our *in vitro* angiogenic model also provides a niche for HSPCs, we established vessel networks for 14 d before the addition of purified UCB CD34^+^CD133^+^ HSPCs. After a further culture period of 12 d in basic haematopoietic culture media (Myelocult) without addition of cytokines, cells were harvested, counted and their immunophenotype assessed by flow cytometry. Compared to the input, co‐culture of HSPCs with hBMSC alone, or hBMSC and BMEC resulted in a 3·6‐ to 4·0‐fold increase in total viable nucleated cells (Figure S2A; *P ≤ 0·05) and the total number of CD45^+^CD34^+^CD133^+^ cells compared to that of the input was not significantly reduced when HSPCs were co‐cultured with hBMSC and BMEC (Figure S2B; *P* = 0·20), despite rapid attrition of HSPCs in conditions without additional cytokines or supportive cells. We also found that key haematopoietic cytokines, such as CXCL12 and SCF, known to affect HSPC fate and retention within the bone marrow niche *in vivo* (Barker, 1994, 1997; Wright *et al*, 2005), were also expressed in our model (Figure S2E). Taken together, these data indicate that our *in vitro* model of the niche not only supports angiogenesis, but also provides a niche for maintaining HSPCs.

### Differential expression of pro‐angiogenic proteins in human bone marrow stromal cells and human dermal fibroblasts

**Figure 2:**
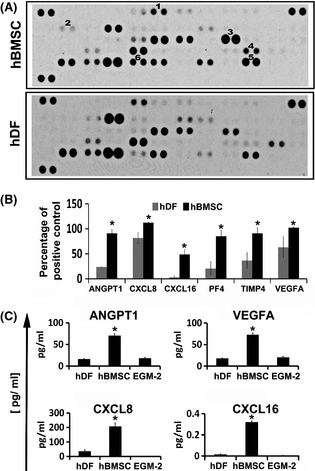
Differential expression of pro‐angiogenic proteins in human bone marrow stromal cells and human dermal fibroblasts (hDF). Conditioned media from two separate batches of human bone marrow stromal cells (hBMSC) and hDF were analysed using angiogenic antibody arrays. Representative blots are shown in (A; 1–6 = ANGPT1, CXCL16, CXCL8, PF4, TIMP4 and VEGFA respectively). The mean pixel density was calculated using ImageJ software for each protein tested and the relative percentage of mean pixel density for each protein was calculated after subtraction of the negative control (B). Quantitative ELISAs were performed on the conditioned medium obtained from hBMSC and hDF to compare the levels of ANGPT1, VEGFA, CXCL16 and CXCL8 (C). Results were analysed using Student's *t*‐test, *P ≤ 0·05. Data are mean ± standard deviation of at least three separate experiments using at least two separate batches of hDF and hBMSC. EGM‐2, Endothelial Cell Growth Medium‐2.

It is acknowledged that it is difficult to distinguish between different stromal cell sources by phenotypic characterization (Blasi *et al*, 2011). Indeed, we found that hBMSC and hDF displayed similar immune‐phenotypes, but rather different ability to support blood vessel formation by BMEC. Therefore, we next determined the angiogenic profiles of hBMSC and hDF. To this end, we performed angiogenic antibody arrays to compare the relative expression of 55 known human angiogenic proteins in hBMSC *versus* hDF cells. Figure 2A shows representative blots of the angiogenic arrays from either hBMSC or hDF CM. We identified a total of six factors with significantly and consistently higher expression levels in CM collected from all hBMSC batches, compared to all hDF batches used (*P* ≤ 0·05; Fig 2B). Using this method, we identified significant differences (*P* ≤ 0·05) in the anti‐angiogenic factors, platelet factor 4 (PF4) and tissue inhibitor of metalloproteinase 4 and also in the pro‐angiogenic factors VEGFA, ANGPT1, CXCL8 and chemokine (C‐X‐C motif) ligand 16 (CXCL16). In order to confirm the results of the angiogenic array profiling, we quantified the level of pro‐angiogenic factors in the CM of hDF and hBMSC and found significantly higher levels of ANGPT1, VEGFA, CXCL8 and CXCL16 (*P* ≤ 0·05; *c*. 4·2‐fold, 4‐fold, 18·6‐fold and 5·6‐fold, respectively) in hBMSC CM than in hDF CM by ELISA (Fig 2C).

### CXCL8/CXCR2 modulates vessel formation of human bone marrow endothelium *in vitro*

**Figure 3:**
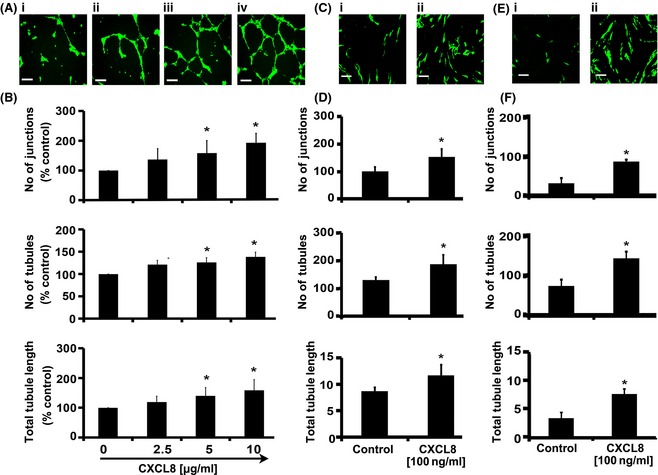
CXCL8 increases vessel formation of human bone marrow endothelial cells. Bone marrow endothelial cells (BMEC) were seeded onto growth factor‐reduced Matrigel and supplemented with increasing doses of cytokine or carrier (0·5% w/v bovine serum albumin) as indicated in the figure. Representative pictures (A: i–iv) of vessel networks formed in Matrigel in response to CXCL8 (scale bars = 200 μmol/l) and (B) quantification of number of junctions, number of tubules, and total tubule length after 16 h incubation are shown. Representative images of vessel formation are also shown from co‐cultures of BMEC with either human bone marrow stromal cells (hBMSC) or human dermal fibroblasts (hDF) incubated in complete EGM‐2 media supplemented with 8% (v/v) fetal calf serum and either carrier or CXCL8 (C: i & ii and E: i & ii respectively; scale bars = 500 μmol/l), Vessel networks were visualized by fluorescence microscopy of eGFP‐labelled BMEC. Quantification of number of junctions (branch points), number of tubules and total tubule length in co‐cultures of BMEC and hBMSC (D) or BMEC and hDF (F) in the presence of carrier or CXCL8 are also shown. Results were analysed using Student's *t*‐test, *P ≤ 0·05. Data are mean ± standard deviation of at least three separate experiments.

Cytokines, such as ANGPT1 and VEGFA, have been implicated in the angiogenic/haematopoietic repair of the murine bone marrow endothelium following pre‐conditioning prior to transplantation *in vivo* (Kopp *et al*, 2005; Hooper *et al*, 2009). However, the pro‐angiogenic effect of CXCL8 on human bone marrow endothelium has not been tested previously. We used the reduced cytokine Matrigel assay, an assay that permits testing the effect of an individual cytokine with minimal disturbances from other cytokines. Figure 3A shows representative images of BMEC after 16 h supplementation with increasing concentrations of CXCL8 (Fig 3A: i–iv). Quantification of vessel network parameters revealed that treatment of BMEC with CXCL8 produced a significant increase (Fig 3B; *P* ≤ 0·05) in the number of junctions and tubules and the total tubule length compared to untreated cells. We also found a significant increase in transwell migration (*c*. 1·5‐fold) of BMEC in response to CXCL8 (Figure S3A; *P* ≤ 0·05). In order to test if CXCL8 could also induce vessel network formation in our bone marrow co‐culture assay, we used eGFP‐labelled BMEC cells and co‐cultured them with hBMSC for 14 d in the absence or presence of CXCL8 (Fig 3C: i and ii, respectively). The addition of CXCL8 to the BMEC/hBMSC co‐cultures also significantly increased the number of junctions, tubules and total tubule length (Fig 3D). In co‐cultures of hDF and BMEC, the addition of CXCL8 also increased blood vessel formation (Fig 3E and F). Taken together, these data confirm that CXCL8 has a pro‐angiogenic effect upon human BMEC *in vitro*.

**Figure 4:**
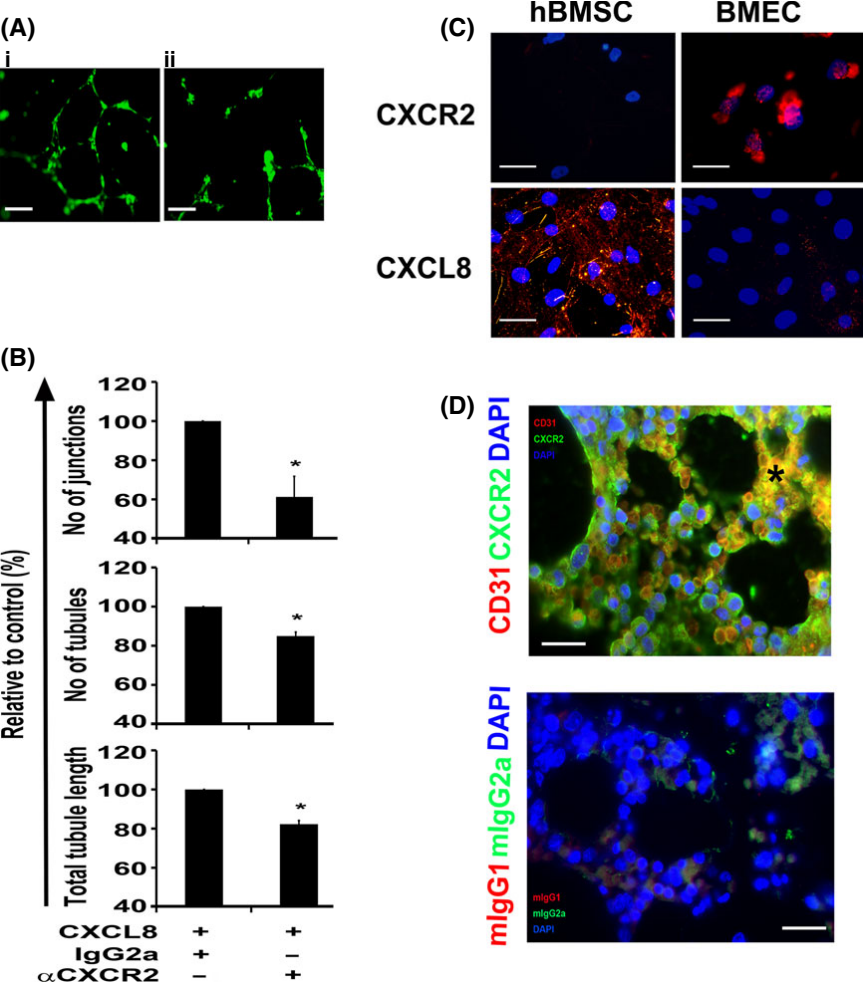
CXCR2 modulates angiogenesis and is expressed on human bone marrow endothelium. eGFP‐labelled human bone marrow endothelial cells (BMEC) were seeded onto growth factor‐reduced Matrigel and supplemented with CXCL8 (5 μg/ml) in the presence of either CXCR2 neutralizing antibody (20 μg/ml) or isotype control (IgG2a, 20 μg/ml). Representative pictures of vessel networks formed after 16 h either in presence of isotype control or anti‐CXCR2 neutralizing antibody are shown (A: i and ii respectively). Quantification of number of junctions (branch points), number of tubules and total tubule length in the absence or presence of CXCR2 neutralizing antibody (αCXCR2) is shown (B). Representative pictures of BMEC or hBMSC stained with either CXCR2 or CXCL8 (C; scale bars = 200 μm) and human normal bone marrow biopsies co‐stained with anti CD31 (red) and CXCR2 (green) antibodies (D; *sinusoid, scale bars = 50 μm). Cell nuclei were stained with DAPI (blue). Results were analysed using Student's *t*‐test, *P ≤ 0·05. Data are mean ± standard deviation of at least three independent experiments.

Previous studies have shown that blocking the CXCR2 receptor can inhibit tumour growth, cell invasion and angiogenesis (Singh *et al*, 2010; Nannuru *et al*, 2011). In order to test if CXCR2 is mechanistically important in CXCL8‐mediated angiogenesis of BMEC, we cultured BMEC on growth factor reduced Matrigel in the presence of CXCL8 and CXCR2 neutralizing antibody or isotype control. Figure 4A shows representative images of reduced vessel formation in CXCL8 treated BMEC in the presence of CXCR2 neutralizing antibody (Fig 4A: ii) but not the control antibody (Fig 4A: i). The number of junctions, vessels and total tubule length were all significantly reduced in the anti‐CXCR2‐treated BMEC compared to control (Fig 4B; *P* ≤ 0·05). Additionally, we found that the addition of CXCR2 neutralizing antibody also significantly reduced vessel formation in our hBMSC/BMEC co‐culture assay (Figure S4B). Next, we tested the expression of CXCL8 and CXCR2 in both BMEC and hBMSC (Fig 4C) and CXCR2 in normal human bone marrow trephine biopsies (Fig 4D). We found that CXCL8 was highly expressed in hBMSC and not significantly in BMEC, whilst CXCR2 was expressed in BMEC but not hBMSC. CXCR2 was co‐expressed in CD31‐positive regions of human bone marrow, including expression in morphologically recognizable bone marrow endothelium in vascular structures *in situ* (Fig 4D: *). These data indicate that CXCR2 is expressed in human bone marrow sinusoids and is mechanistically important in CXCL8‐mediated angiogenesis.

### Haematopoietic reconstitution is delayed in Cxcr2 knockout mice due to impaired angiogenic repair

**Figure 5:**
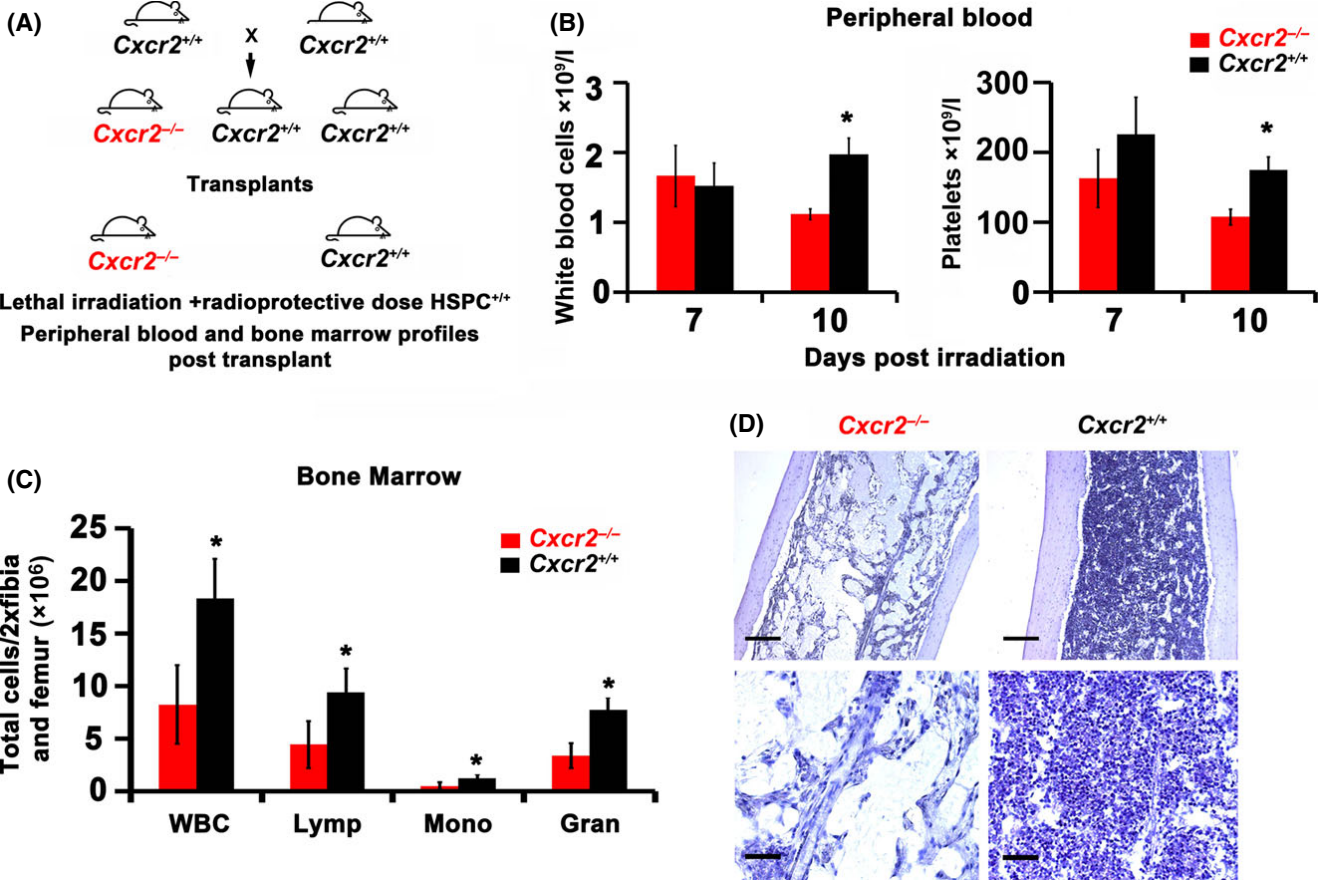
Delayed haematopoietic reconstitution in *Cxcr2* knockout animals. B6.129S2(C)‐*Cxcr2* ^*m1Mwm*^/J mice, heterozygous for *Cxcr2* (*Il8rb)* knockout were obtained from Jackson Laboratories and bred in house to generate *Cxcr2* homozygous knockout and littermate wild type controls. *Cxcr2* homozygous knockout (−/−) and wild type (+/+) littermate control animals were treated with 9·5 Gy irradiation dose and transplanted within 24 h with a radio‐protective dose (1 × 10^6^) of total wild type bone marrow cells. Peripheral blood samples were taken from animals at days 7 and 10 and haematology analysis performed on a veterinary blood analyser. After 14 d femurs and tibias were harvested and bone marrow extracted before performing haematology analysis. Schematic representation of study (A), comparative white blood cell and platelet counts in peripheral blood (B), bone marrow haematology analysis at day 14 (WBC = white blood cell count, Lymp = lymphocyte, Mono = monocyte and Gran = granulocyte; C), and representative images of low magnification (top panel; scale bars = 200 μm) and higher magnification (bottom panel; scale bar = 50 μm) bone marrow sections stained with haematoxylin to reveal cellularity (D). Data are mean ± standard deviation. *n* = 13–16 animals per group. Results were analysed using Student's *t*‐test, *P ≤ 0·05 comparing cell numbers in *Cxcr2* ^−/−^ *versus* wild type (*Cxcr2* ^+/+^) animals.

CXCL8 increases human endothelial cell permeability in the early stages of angiogenesis and can trans‐activate VEGFR2 (KDR) through dimerization of CXCR2 with VEGFR2 (Petreaca *et al*, 2007). Hooper *et al* (2009) showed that VEGFR2 modulates angiogenesis and thereby haematopoiesis in murine models of engraftment. We therefore hypothesized that deficiency in CXCR2 may also impair endothelial recovery and haematopoietic reconstitution. *Cxcr2* ^*+/−*^ mice were crossbred to generate *Cxcr2* ^*+/+*^ (wild type (wt)) and *Cxcr2* ^−/−^ (knockout) littermates (Fig 5A). To induce severe vascular regression in the bone marrow, both wild type (wt) and *Cxcr2* ^−/−^ mice were challenged with a lethal (9·5 Gy) dose of radiation and then transplanted with a radio‐protective dose of bone marrow cells. Peripheral blood profiles were determined by haematology analysis in transplanted animals at day 4, 7, 10 and 14 post‐irradiation. Total bone marrow cellularity was also determined at day 14.

As expected, both platelet counts and white blood cell (WBC) counts in the peripheral circulation decreased dramatically from day 4 to 7 post‐irradiation and transplantation (1009 ± 129 × 10^9^ platelets/l and 4·85 ± 1·64 × 10^9^ WBC/l to 139 ± 47·8 × 10^9^ platelets/l and 4·85 ± 1·64 × 10^9^ WBC/l for *Cxcr2* ^−/−^ and from 848 ± 88·9 × 10^9^ platelets/l and 4·75 ± 1·18 × 10^9^ WBC/l to 226 ± 47·5 × 10^9^ platelets/l and 1·52 ± 0·33 × 10^9^ WBC/l for *Cxcr2* ^*+/+*^; Fig 5B). By day 10, compared to wt littermate controls, *Cxcr2* ^−/−^ mice displayed significantly lower WBC and platelet counts (Fig 5B) in the peripheral blood following transplantation (*P* ≤ 0·05). Bone marrow haematology profiling and histology at day 14 also revealed a significant reduction in the total WBC count and in lymphocytes, monocytes and granulocytes (Fig 5C, *P ≤ 0·05) and a grossly reduced bone marrow cellularity (Fig 5D) in *Cxcr2* ^−/−^ mice compared to wt. However, despite persistent delays in bone marrow recovery in *Cxcr2* ^−/−^ mice, by day 14 there was no significant difference in peripheral blood profiles between *Cxcr2* ^−/−^ and wt animals (265 ± 83 × 10^9^ platelets/l and 3·20 ± 0·99 × 10^9^ WBC/l and 305 ± 1·31 × 10^9^ platelets/l and 3·7 ± 1·31 × 10^9^ WBC/l and for *Cxcr2* ^−/−^ and *Cxcr2* ^+/+^; *P* = 0·45 and 0·66 respectively).

**Figure 6:**
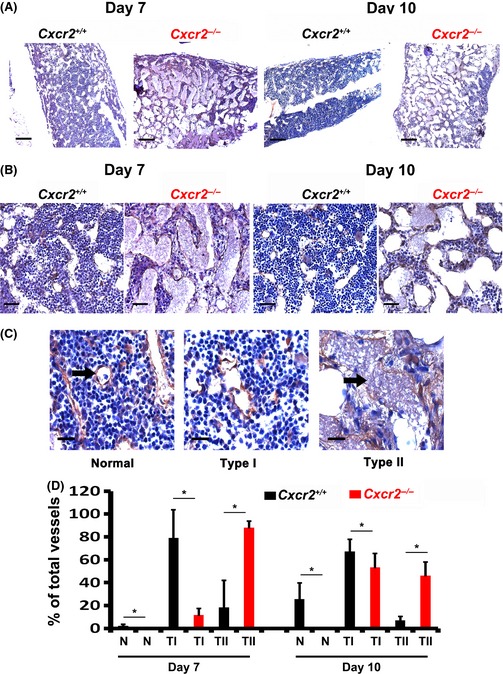
Impaired vascular regeneration in *Cxcr2* knockout animals. B6.129S2(C)‐*Cxcr2* ^*tm1Mwm*^/J mice, homozygous knockout (*Cxcr2* ^−/−^) or wild type (*Cxcr2* ^+/+^) littermates were treated with 9·5 Gy irradiation dose and transplanted within 24 h with a radio‐protective dose (1 × 10^6^) of total bone marrow cells. After 7 or 14 d femurs were harvested, fixed and embedded in paraffin. Bone marrow sections were stained with anti‐mouse VE‐Cadherin antibody to highlight bone marrow endothelium. Representative images of recovering bone marrow endothelium at low magnification (A; scale bars = 200 μm) and higher magnification (B; scale bars = 50 μm) post‐irradiation are shown. Representative images of examples of normal (N), or type I (mild/moderately damaged) or type II (significantly damaged) bone marrow vessels (C; scale bars = 25 μm; black arrow) and quantification of these vessel types post‐recovery are shown (D). Data are mean ± standard deviation. *n* = 5 animals per group. Results were analysed using analysis of variance (anova) and Student's *t*‐test, *P ≤ 0·05 comparing vessel numbers in *Cxcr2* ^−/−^ *versus* wild type (*Cxcr2* ^+/+^) animals.

We hypothesized that delays in bone marrow haematopoietic recovery would be preceded by delayed bone marrow angiogenic repair at earlier time points. Therefore, we harvested femurs from irradiated and transplanted animals at days 7 and 10 post‐irradiation and examined bone marrow sections to assess the degree of endothelial repair/regeneration. Figure 6A and B represent low and high magnification images of bone marrow at day 7 and 10 post‐irradiation showing gross histological differences between wt and *Cxcr2* ^−/−^ littermates. In order to quantify the endothelial repair more precisely, we used the method developed by Hooper *et al* (2009), and scored random bone marrow fields for either normal (N), type I (mild/moderately damaged) or type II (significantly damaged) vessels. Representative examples of high magnification images of normal and progressively more damaged type I and type II vessels are shown in Fig 6C. Figure 6D shows quantification of normal, type I and type II vessels at days 7 and 10 post‐irradiation injury in wt and *Cxcr2* ^−/−^ mice. At day 7 post‐irradiation there were very few normal vessels in either the wt or *Cxcr2* ^−/−^ animals, with normal vessels comprising <5% of those quantified in each field. However, when damaged vessels are quantified, significant differences in the percentage of type I and II vessels are revealed; whilst type I vessels comprised approximately 80% of the vessel types in the wild type littermate controls, *Cxcr2* ^−/−^ animals displayed only approximately 10% type I vessels. *Cxcr2* ^−/−^ animals were found to have significantly higher percentages of type II vessels at day 7 post‐irradiation (*c*. 90%) than the *Cxcr2* ^+/+^ mice (*c*. 15%; *P* ≤ 0·05), indicating that there is more endothelial damage in the bone marrow of *Cxcr2* ^−/−^ animals. The bone marrow of animals at day 10 post‐irradiation also revealed significant differences between wt and *Cxcr2* ^−/−^ animals. Histologically, the wt littermate controls still appeared grossly different at this time point (Fig 6A and B) and quantification revealed significantly higher numbers of normal vessels in the wt animals compared to the *Cxcr2* ^−/−^ animals (representing about 25% compared to <5% of total vessels respectively; *P* ≤ 0·05). In terms of type I and type II damaged vessels, we observed no significant difference between the percentage of type I vessels between groups, however, we found significantly higher numbers of type II (more damaged) vessels in the bone marrow of the *Cxcr2* ^−/−^ animals compared to the wt littermate controls (*c*. 10% vs. *c*. 50% respectively; *P* ≤ 0·05). No significant differences in the number of bone marrow vessels were observed in *Cxcr2* ^−/−^ animals or wt littermates could be found prior to myeloablation (Figure S4A and B). However, and as previously described (Cacalano *et al*, 1994), we found significantly higher numbers of nucleated cells in the bone marrow of *Cxcr2* ^−/−^ mice compared to wt littermates under normal steady‐state conditions (Figure S4C). Also, following myeloablation, we found no significant difference in the number of viable sinusoidal endothelial cells in the bone marrow of wt compared to *Cxcr2* ^−/−^ mice at day 4 by flow cytometric analysis (Figure S4D). Taken together, these data suggest that loss of *Cxcr2* function results in impaired bone marrow endothelial repair, which ultimately results in delayed bone marrow haematopoietic reconstitution following BMT.

## Discussion

Given the rapidly emerging clinical significance of bone marrow endothelium in both health and disease and the scarcity of suitable humanized, tissue‐specific models to quantify angiogenesis and associated factors in the human bone marrow endothelial niche, we set out to recreate such a model in our laboratory. In this study, we have successfully generated an *in vitro* model of the human bone marrow endothelial niche, which can be used to study angiogenesis. Whereas other models of the bone marrow niche have been established (de Barros *et al*, 2010; Carrion *et al*, 2010; Li *et al*, 2011; Saleh *et al*, 2011), to our knowledge these do not contain both stromal and endothelial cells specifically derived from human bone marrow. We demonstrated that human BMEC could consistently form blood vessel networks when co‐cultured with hBMSC, while hDF failed to support BMEC vessel networks. When co‐cultured with HSPCs with Myelocult and no additional cytokine addition, our hBMSC/BMEC model also supported a small, but significant, expansion of total viable nucleated cells (3·6‐ to 4·0‐BMSCfold). Notably, CD45^+^CD133^+^CD34^+^ HSPC numbers could not be maintained with hBMSCs but required addition of BMECs. These data generated in our humanized system are in line with other studies that demonstrate the importance of the murine bone marrow endothelial niche in HSC maintenance (Ding & Morrison, 2013; Greenbaum *et al*, 2013).

The hBMSC used in this study express a typical stromal cell phenotype similar to that reported of hDF. It is also noted by us and others that immunophenotyping alone may not easily distinguish differences in cellular composition (Blasi *et al*, 2011) in these cell types. We therefore examined whether differences in the vascular supportive ability of hBMSC compared to hDF may be due to differential expression of angiogenic secreted factors. The present study demonstrated that six angiogenic factors were differentially expressed in hBMSC and hDF, highlighting the importance that tissue‐specific cues may have in generating new blood vessels. Interestingly, ANGPT1 and VEGFA have been shown to act co‐operatively in modulating the angiogenic repair of damaged murine bone marrow sinusoidal endothelium in murine models of transplantation *in vivo* (Kopp *et al*, 2005). Although more extensive studies of protein analysis are likely to reveal further significant differences between hDF and hBMSC, and the angiogenic factors described here are not uniquely expressed in bone marrow, we hypothesized that the up‐regulation of ANGPT1, VEGFA, CXCL16 and CXCL8 may contribute to the support of bone marrow angiogenesis and recovery. To this end, we demonstrated that CXCL8 increased vessel formation of BMECs in the reduced growth factor Matrigel system and also BMEC migration. Additionally, blocking CXCL8 signalling with neutralizing antibodies targeting CXCR2 significantly reduced the vascular networks formed by BMEC, both in our co‐culture assay and in response to CXCL8 in Matrigel. To our knowledge, this is the first time that CXCL8 has been directly implicated in the angiogenic process of the human bone marrow system, and this may have future clinical applications.

CXCL8 promotes human endothelial cell permeability in the early stages of angiogenesis and trans‐activates VEGFR2, in a VEGFA‐independent manner, by causing the dimerization of VEGFR2 with CXCR2 (Petreaca *et al*, 2007). It has also been demonstrated by others that *Cxcr2* ^−/−^ mice have impaired skin revascularization following injury (Devalaraja *et al*, 2000; Milatovic *et al*, 2003). Therefore, we used *Cxcr2* ^−/−^ transgenic mice to test our hypothesis that CXCR2 plays a role in the repair and regeneration of bone marrow endothelium and therefore haematopoietic recovery *in vivo*. Through this, we have demonstrated for the first time that CXCR2 loss of function delays the repair and regeneration of bone marrow endothelium and bone marrow haematopoietic recovery following myeloablation and BMT. In lethally irradiated mice Hooper *et al* (2009) convincingly demonstrated that the period of 7–10 d post‐irradiation is critical for both vascular regeneration and haematopoietic recovery, with VEGFA and VEGFR2 playing an important role in those processes. We observed similar recovery kinetics in *Cxcr2* ^−/−^ myeloablated mice and comparable levels of vascular and haematopoietic impairment at early time points. However, whilst our *Cxcr2* ^−/−^ animals survived longer term, *Kdr* (*Vegfr2)* knockout animals used by others did not (Hooper *et al*, 2009). In fact, despite persistent delays in bone marrow recovery in *Cxcr2* ^−/−^ mice, by day 14, there was no longer a significant difference in peripheral blood profiles of *Cxcr2* ^−/−^ and *Cxcr2* ^+/+^ animals. This may simply be explained by our transplantation protocol, where, for welfare reasons, we have administered double the dose of donor bone marrow, or may indicate other compensatory mechanisms, such as extra medullary haematopoiesis. There is no doubt that, during the complex process of repair and regeneration, significant interplay between haematopoietic and angiogenic factors will be modulating both haematopoiesis and angiogenesis in the bone marrow (Bikfalvi & Han, 1994) and it is unlikely to be explained simply by a single factor. However, in this study we show that CXCR2 contributes to the process of repair and regeneration within the bone marrow endothelial niche.

In summary, we have developed for the first time an *in vitro* model of the human bone marrow endothelial niche consisting of cells derived entirely from human bone marrow tissue that permits accurate quantification of angiogenesis and have demonstrated the importance of CXCL8 as a pro‐angiogenic factor in this model. In addition, we have also shown, for the first time, a role of CXCR2 in bone marrow angiogenesis and haematopoietic recovery. We hope that this study will have important future clinical implications for improving haematopoietic recovery following BMT.

## Disclosure of conflicts of interest

The authors declare no conflict of interest.

## Author contributions

SJMH raised funding, conceived ideas, discussed the hypothesis, participated in the design of all experiments, performed all experiments, analysed and interpreted data and prepared the manuscript and figures; ABHH participated in experimental design of and assisted with transplantation experiments. YZ assisted with the niche assay and histology experiments. DS assisted with haematology analysis. MVG assisted with transplantation experiments, RG assisted with IHC and data analysis. EP performed phenotyping analysis of hBMSC. NF and KC provided essential resources. EMR raised funding, conceived ideas, proposed hypothesis and design project, analysed and interpreted the data, directed the project and prepared the manuscript and figures. SMW raised funding, conceived ideas, participated in experimental design, interpreted the data, directed the project and critically reviewed the manuscript. All authors approved the final manuscript.

## Supporting information


**Fig S1.** Human bone marrow endothelial cells form quantifiable vessel networks when co‐cultured with primary bone marrow stromal cells.

**Fig S2.** Human bone marrow endothelial cells and human bone marrow stromal cells co‐cultures provide a niche for haemopoietic stem and progenitor cells.

**Fig S3.** Human bone marrow endothelial cells migrate in response to CXCL8.

**Fig S4.** Bone marrow endothelial vessels in steady state and post irradiation in Cxcr2^−/−^ and wild type animals.
