# Supplementary figures and images for: CXCR2 modulates bone marrow vascular repair and haematopoietic recovery post‐transplant
Source: Br J Haematol. 2015 Mar 9;169(4):552–64. doi: 10.1111/bjh.13335 (PMC4654909; doi:10.1111/bjh.13335)

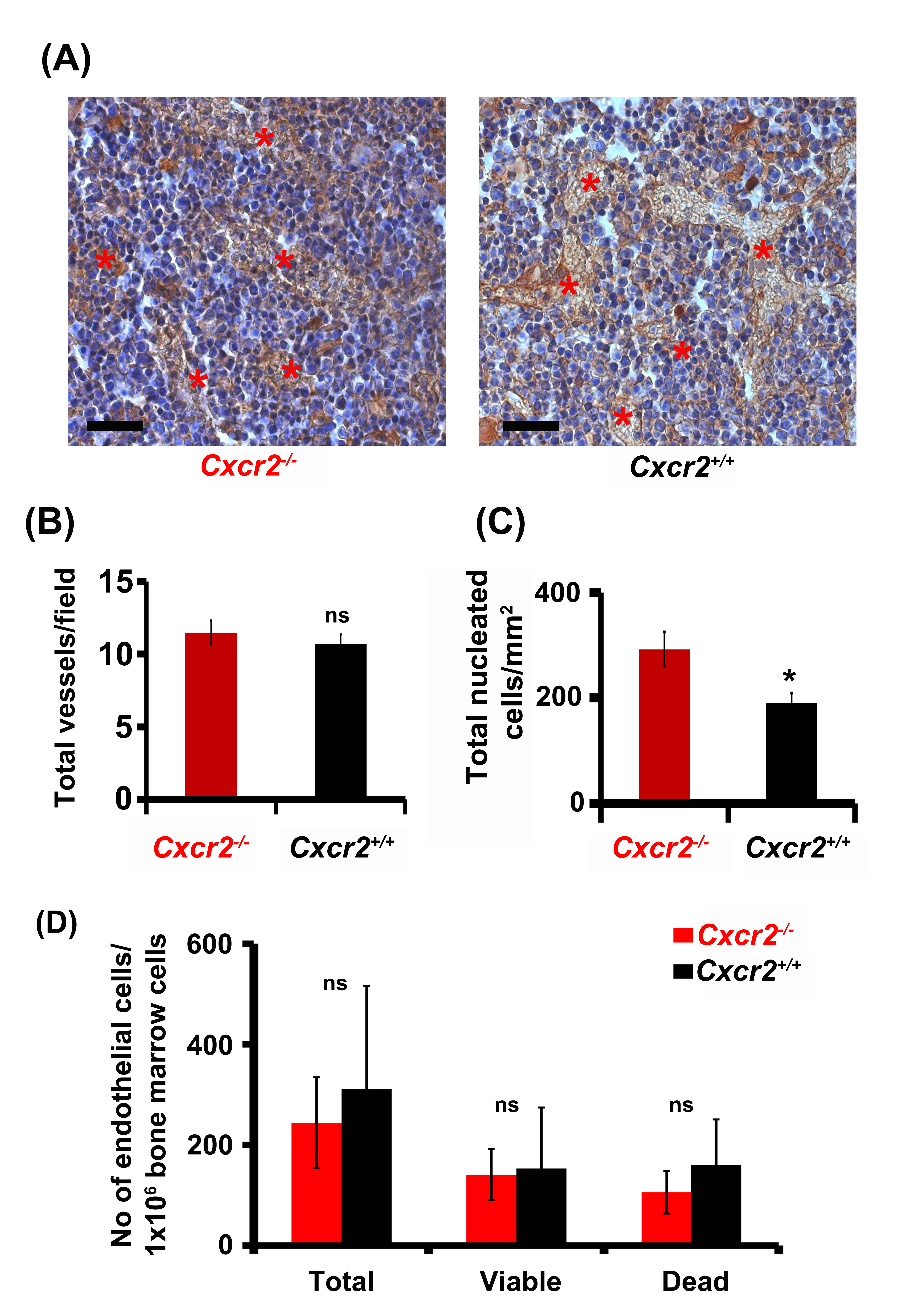

Supplement: Supplementary file 4 — Fig S4. Bone marrow endothelial vessels in steady state and post irradiation in Cxcr2−/− and wild type animals. [file bjh0169-0552-sd4.tif]
